# Emergence of high-level colistin resistance mediated by multiple determinants, including *mcr-1.1*, *mcr-8.2* and *crrB* mutations, combined with tigecycline resistance in an ST656 *Klebsiella pneumoniae*

**DOI:** 10.3389/fcimb.2023.1122532

**Published:** 2023-01-26

**Authors:** Yanfei Wang, Junxin Zhou, Haiyang Liu, Qian Wang, Ping Zhang, Jingyi Zhu, Dongdong Zhao, Xueqing Wu, Yunsong Yu, Yan Jiang

**Affiliations:** ^1^ Department of Infectious Diseases, Sir Run Run Shaw Hospital, Zhejiang University School of Medicine, Hangzhou, China; ^2^ Key Laboratory of Microbial Technology and Bioinformatics of Zhejiang Province, Hangzhou, China; ^3^ Regional Medical Center for National Institute of Respiratory Diseases, Sir Run Run Shaw Hospital, Zhejiang University School of Medicine, Hangzhou, China; ^4^ Department of General Practice, Sir Run Run Shaw Hospital, Zhejiang University School of Medicine, Hangzhou, China

**Keywords:** colistin, *mcr*, tigecycline, *tmexCD1*-*toprJ1*, co-integration

## Abstract

Colistin and tigecycline are usually regarded as the last resort for multidrug-resistant *Klebsiella pneumoniae* infection treatment. Emergence of colistin and tigecycline resistance poses a global healthcare challenge and is associated with high mortality due to limited therapeutic options. Here, we report the ST656 extensively drug-resistant *K. pneumoniae* strain KP15-652, which was isolated from a patient’s urine in China. Antimicrobial susceptibility testing showed it to be resistant to tigecycline, amikacin, levofloxacin, ciprofloxacin, and high-level colistin resistance (> 2048 mg/L). Whole-genome sequencing revealed that it harbors one chromosome and seven plasmids, including four plasmids carrying multiple acquired resistance genes. Transformation/conjugation tests and plasmid curing assays confirmed that *mcr-1.1*, *mcr-8.2* and *crrB* mutations are responsible for the high-level colistin resistance and that a series of efflux pump genes, such as *tmexCD1-toprJ1*, *tet*(A) and *tet*(M), contribute to tigecycline resistance. *mcr-1.1* and *tet*(M) are located on an IncX1 plasmid, which has conjugation transfer potential. *mcr-8.2* and *tet*(A) are located on a multireplicon IncR/IncN plasmid but unable to be transferred *via* conjugation. Moreover, another conjugable and fusion plasmid carries the *tmexCD1-toprJ1* gene cluster, which may have arisen due to IS*26*-mediated replicative transposition based on 8-bp target-site duplications. Importantly, a complex class 1 integron carrying various resistance genes was detected on this fusion plasmid. In conclusion, it is possible that the high-level of colistin resistance is caused by the accumulated effect of several factors on the chromosome and *mcr*-carrying plasmids, combined with many other resistances, including tigecycline. Effective surveillance should be performed to prevent further dissemination.

## Introduction

*Klebsiella pneumoniae* is one of the most common opportunistic pathogens. It usually causes various hospital-acquired infections, including respiratory tract infections, urinary tract infections, and bloodstream infections, posing an emerging challenge for clinical settings worldwide (Wyres et al., 2020b; Wareth and Neubauer, 2021; Gorrie et al., 2022). Importantly, the emergence and spread of extensively drug-resistant *K. pneumoniae* (XDR-KP) severely limits the effective use of antimicrobial agents (Jin et al., 2021). Thus, treatment options for XDR-KP mainly rely on the last-resort antibiotics colistin and tigecycline. However, *K. pneumoniae* may develop a succession of resistance-associated determinants, including related chromosomal-located mutations or acquired plasmid-mediated resistance genes (Liu et al., 2021).

Colistin is a kind of active agent against life-threatening infections by XDR-KP (El-Sayed Ahmed et al., 2020). Unfortunately, misuse of colistin in hospitalized patients and animals in stock farming has led to the emergence of colistin-resistant XDR-KP. The underlying mechanisms contributing to colistin resistance in XDR-KP are usually mediated by chromosomal mutations (*mgrB*, *phoP*/*phoQ*, *pmrA*/*pmrB* and *crrAB*) as well as plasmid-related *mcr-1* and its variants (*mcr-2* to *mcr-10*) (Liu et al., 2016; Jayol et al., 2017; Poirel et al., 2017; Wang et al., 2018). Nonetheless, the co-existence of *mcr-1* and *mcr-8* in XDR-KP is relatively rare (Liu et al., 2021).

Tigecycline is also deemed one of the few drugs of choice for treating XDR-KP infection. Regardless, tigecycline-resistant Enterobacteriaceae are increasingly being reported following widespread clinical use of this antibiotic. The mechanisms of resistance to tigecycline are related to mutations in ribosome (*rpsJ* gene) (Beabout et al., 2015) and plasmid-mediated mobile resistance genes (*tet*(X) variants, *tet*(A) and *tet*(M) genes) (Fiedler et al., 2016; Linkevicius et al., 2016; Sun et al., 2019; Lv et al., 2020 Xu et al., 2021; Wang et al., 2021c). Strikingly, a kind of plasmid-located resistance-nodulation-division (RND) efflux pump, namely *tmexCD1*-*toprJ1*, that could confer resistance to tigecycline was identified in *K. pneumoniae* in 2020 in China (Lv et al., 2020; Sun et al., 2020). To date, emergence of *tmexCD1*-*toprJ1* gene cluster and its variants are increasing in stock farming or nosocomial samples (Wang et al., 2021a; Wang et al., 2021b; Gao et al., 2022), and the genetic structure characteristics of these plasmid-mediated tigecycline resistance determinants raised a major concern worldwide (Dong et al., 2022).

In this study, we characterized the whole genome structure of an ST656 XDR *K. pneumoniae* strain harboring *mcr-1.1*, *mcr-8.2*, and *crrB* mutations and the *tet*(A), *tet*(M), and *tmexCD1*-*toprJ1* gene cluster located on chromosome or several distinct plasmids. The *tmexCD1*-*toprJ1*-carrying fusion plasmid that formed through IS*26-*mediated co-integration events was further analyzed completely from an evolutionary perspective.

## Materials and methods

### Bacterial isolation and identification

The KP15-652 strain was isolated by urine culture from a patient with a trauma to the urinary track in Quzhou, China on October 20, 2015. Matrix-assisted laser desorption ionization–time of flight mass spectrometry (MALDI-TOF MS, BioMeírieux, France) was used to identify the isolate to the species level and further confirmed using genome sequencing data.

### Antimicrobial susceptibility testing

Minimum inhibitory concentrations (MICs) of the KP15-652 strain for cefepime, cefotaxime, imipenem, meropenem, amikacin, levofloxacin, ciprofloxacin, fosfomycin, aztreonam, colistin and tigecycline were determined by broth microdilution. The results were interpreted according to Clinical and Laboratory Standards Institute (CLSI) 2021 guidelines. *Escherichia coli* ATCC 25922 served as the quality control strain.

### Whole-genome sequencing and analysis

Genomic DNA of the KP15-652 strain was extracted using a Qiagen Minikit (Qiagen, Hilden, Germany) based on the manufacturer’s recommendations. Whole-genome sequencing was performed using both the Illumina NovaSeq platform (Illumina, San Diego, CA, USA) and long-read PacBio RS II platform (Pacific Biosciences, Menlo Park, CA, USA). *De novo* hybrid assembly of the Illumina and PacBio reads was performed using Unicycler v0.4.8 (Wick et al., 2017). Annotation was conducted using National Center for Biotechnology Information (NCBI) Prokaryotic Genome Annotation Pipeline (PGAP) (http://www.ncbi.nlm.nih.gov/genome/annotation_prok/) (Tatusova et al., 2016). Antimicrobial resistance genes were identified using ABRicate v0.8.13 (https://github.com/tseemann/abricate) based on the ResFinder database (http://genomicepidemiology.org/) (Zankari et al., 2012). Bacterial virulence factors were identified using the Virulence Factor Database (VFDB, http://www.mgc.ac.cn/VFs/) (Liu et al., 2022). Capsular typing was performed using *Kaptive* (Wyres et al., 2020a). Plasmid replicons were analyzed with PlasmidFinder v2.1 (Carattoli et al., 2014). Multilocus sequence typing (MLST) was analyzed using the Center for Genomic Epidemiology (CGE) database (https://cge.cbs.dtu.dk/services/MLST/). Insertion sequence (IS) elements were investigated through ISFinder (https://www-is.biotoul.fr/) (Siguier et al., 2006). Genetic contexts were compared with Easyfig (Sullivan et al., 2011). The results of genetic environment and plasmid circular map comparisons were manually visualized in Adobe Illustrator 2020 (https://www.adobe.com/products/illustrator.html).

### Transformation and conjugation assay

Transformation and conjugation assay were performed to determine whether the plasmids carrying *mcr-1.1*, *mcr-8.2* or *tmexCD1*-*toprJ1* gene cluster are transferable. Plasmid DNA was extracted from clinical isolates using a Qiagen Plasmid MidiKit (Qiagen, Hilden, Germany) and then electrotransformed into *E. coli* DH5α. Transformants were selected on Mueller-Hinton (MH) agar plates containing colistin (2 mg/L) or tigecycline (2 mg/L), together with rifampin (700 mg/L). Conjugation experiments using *E. coli* J53 (sodium azide resistant) as the receptor strain were performed using the film mating method (Yang et al., 2021). Conjugants were screened on MH plates containing colistin (2 mg/L) or tigecycline (2 mg/L), together with sodium azide (200 mg/L). The identity of successful transformants or conjugants was confirmed *via* polymerase chain reaction (PCR) for several marker genes, such as resistance genes, and MALDI-TOF MS.

### Plasmid curing assay

Plasmid curing assays were performed using sodium dodecyl sulfate (SDS, 10% w/v, pH 7.4) (El-Mansi et al., 2000). In brief, one colony was selected and grown in 2 mL MH broth overnight culture at 37°C. Then, 100 μL of overnight culture solution was transferred to fresh MH broth containing 10% SDS and incubated at 37°C, followed by spreading on MH agar plates without antibiotics. Plasmid cured colonies were confirmed by PCR and S1 nuclease pulsed field gel electrophoresis (S1-PFGE).

## Results

### Strain and antimicrobial susceptibility testing

**Figure 1:**
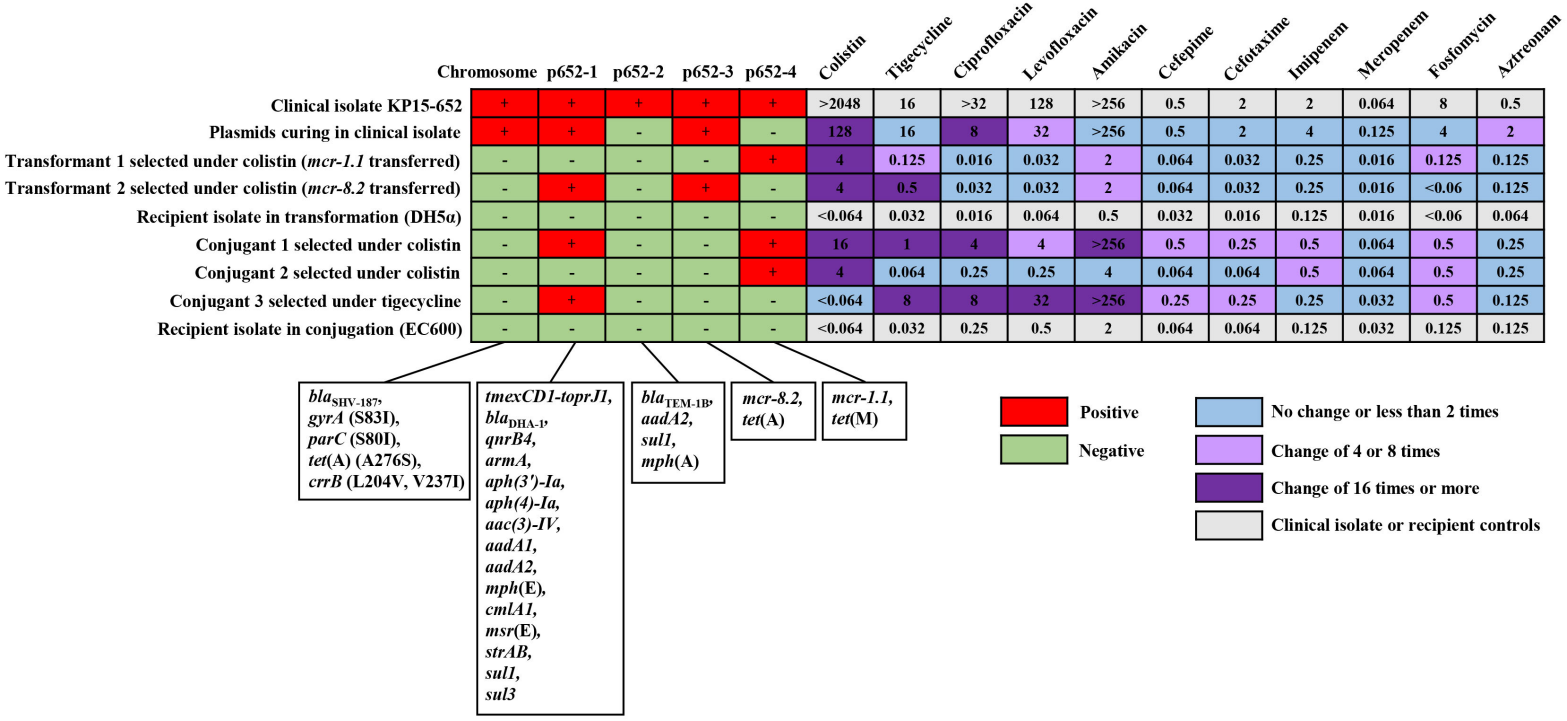
The distribution of resistance determinants on chromosome or plasmids and corresponding MIC manners in clinical isolate, plasmid curing isolate, transformants, conjugants and their recipient controls. For the distribution of resistance determinants, the red cell represents the original chromosome or any positive plasmid, and the green cell represents none. For MICs of the antimicrobial agents, the fold change between any acquired clone in the transferability assay and its relative control is illustrated with blue cells for no change or less than 2 times, light purple cells for change of 4 or 8 times and dark purple cells for change of 16 times or more. The light gray cell indicates MICs of the clinical isolate or recipient controls.

The *K. pneumoniae* strain, named KP15-652, was isolated from urine culture and presented multidrug resistance. MIC data revealed that KP15-652 is resistant to colistin (> 2048 mg/L), tigecycline (16 mg/L), amikacin (> 256 mg/L), levofloxacin (128 mg/L), and ciprofloxacin (> 32 mg/L) but is susceptible to cefepime (0.5 mg/L), cefotaxime (2 mg/L), imipenem (2 mg/L), meropenem (0.064 mg/L), fosfomycin (8 mg/L) and aztreonam (0.5 mg/L). (**Figure 1**) The multidrug resistance and the high-level resistance against colistin prompted us to explore molecular resistance determinants and their transferability based on genomic analysis and phenotype confirmation.

### Genome and molecular typing of KP15-652

The complete genome of isolate KP15-652 was acquired by hybrid assembly combining long- and short-reads sequencing data. The size of the circularized chromosome is 5,265,433 bp (**Table 1**), and up to seven plasmids were identified. MLST analysis showed that the KP15-652 strain belongs to ST656 (*gapA*-*infB*-*mdh*-*pgi*-*phoE*-*rpoB*-*tonB*: 4-4-1-1-7-4-4). The O locus and K locus of KP15-652 are predicted to be OCL101 and KL177, with 94.88% and 99.37% nucleotide identity, respectively.

**Table 1:** Characteristics of KP15-652 genome components.

Location	Replicon	Size (bp)	GC (%)	Resistance genes	Resistance-related Mutations
chromosome	–	5,265,433	57.54%	*bla*_SHV-187_	*gyrA* (S83I), *parC* (S80I), *tet*(A) (A276S), *crrB* (L204V, V237I)
p652-1	IncFIB, IncHI1B	275,345	46.94%	*aph (3’)-Ia*, *strAB*, *aph(4)-Ia*, *aac (3)-IV*, *aadA1* *cmlA1*, *aadA2*, *mph *(E), *msr *(E), *armA*, *sul1*, *sul3*, *bla*_DHA-1_, *qnrB4*, *tmexCD1-toprJ1*	ND
p652-2	IncFIB, IncFII	192,753	52.20%	*aadA2*, *sul1*, *mph *(A), *bla*_TEM-1B_	ND
p652-3	IncR, IncN	52,998	50.17%	*tet *(A), *mcr-8.2*	ND
p652-4	IncX1	43,761	44.57%	*tet* (M), *mcr-1.1*	ND
p652-5	Col440I	3,991	44.35%	ND	ND
p652-6	ColRNAI	3,809	44.58%	ND	ND
p652-7	ColpVC	1,934	51.40%	ND	ND

ND, Not determined.

### Resistance determinants in KP15-652

The KP15-652 genome was positive for a series of antibiotic resistance determinants (**Table 1**, **Figure 1**), conferring potential resistance to β-lactams (*bla*_SHV-187_, blaTEM-1B and *bla*_DHA-1_), aminoglycosides (*strAB*, *aph(3’)-Ia*, *aph(4)-Ia*, *aac(3)-IV*, *aadA1*, *aadA2* and *armA*), macrolide (*mph*(A)*, msr*(E) and *mph*(E)), sulfonamides (*sul1* and *sul3*), quinolones (*qnrB4*), phenicol (*cmlA1*), colistin (*mcr-1.1* and *mcr-8.2*), and tetracycline or tigecycline (*tet*(A), *tet*(M) and *tmexCD1-toprJ1*).

Most of the resistance genes are located on plasmids, especially on the top 4 large plasmids named p652-1, p652-2, p652-3 and p652-4, which are 275,345 bp, 192,753 bp, 52,998 bp and 43,761 bp in size, respectively. The other three plasmids harbored by KP15-652 are relatively small, of no more than 4 kb and carrying no any known resistance genes. Based on replicon analysis, three of four resistant plasmids belong to hybrid incompatibility groups, including IncFIB/IncHI1B for p652-1, IncFIB/IncFII for p652-2 and IncR/IncN for p652-3. The remaining resistant plasmid is from the IncX1 group. The other three small plasmids are rolling-circle replication models belonging to Col440I, ColRNAI, and ColpVC. Plasmid p652-1 harbors a series of resistance genes encoding aminoglycoside-modification enzymes, methylase ArmA, cephalosporinase DHA-1, quinolone resistance determinant QnrB4 and tigecycline efflux pump system tmexCD1-toprJ1. The second large plasmid, p652-2, harbors *aadA2*, *sul1*, *mph*(A) and the β-lactamase gene *bla*_TEM-1B_. *mcr-8.2* is located on plasmid p652-3, accompanied by the wild-type tigecycline efflux pump *tet*(A). Notably, there is another copy of *tet*(A) on the chromosome that shows an A276S mutation, which probably elevates the level of tigecycline resistance. *mcr-1.1* is carried on IncX1 plasmid p652-4 along with another tigecycline efflux pump: *tet*(M) (**Table 1**, **Figure 1**).

Chromosome is also a repository for resistance determinants, such as the β-lactamase gene *bla*_SHV-187_ and tigecycline efflux pump *tet*(A) mutant mentioned above. Moreover, mutations in the *gyrA* (S83I) and *parC* (S80I) genes that confer resistance to fluoroquinolones were detected. Concerning mutations for colistin resistance, mutations in CrrB (L204V, V237I) were identified on the chromosome (**Table 1**, **Figure 1**).

### Transferability of resistance determinants

Due to the concern of resistance to last-resort antibiotics, such as colistin and tigecycline, we performed transformation/conjugation tests and plasmid curing assays to explore the transferability and contribution of corresponding resistance determinants.

Both the transformant and conjugant we acquired under colistin selection presented two distinct phenotypes when we extracted plasmids from a clinical isolate for transformation or used a clinical isolate as a donor for the conjugation assay. One of the phenotypes in each assay involved elevated colistin resistance to more than 64 times compared to the respective recipient due to acquisition of the *mcr-1.1*-harboring plasmid p652-4. The transformation assay particularly selected another phenotype under colistin, which was confirmed as having acquired two plasmids, *tmexCD1-toprJ1*-harboring p652-1 and *mcr-8.2*-harboring p652-3, simultaneously. This transformant showed more than 64 times increased colistin resistance and 16 times higher tigecycline resistance. In addition, another conjugant was captured with co-transfer of plasmids p652-1 and p652-4, resulting in both higher colistin and tigecycline resistance in a similar manner (**Figure 1**).

When tigecycline was used as a selection marker, only a p652-1-harboring conjugant was acquired, with a much higher tigecycline resistance level (MIC increased 256 times). Unfortunately, several attempts for transformation under tigecycline failed. We also attempted the plasmid curing assay using a clinical isolate and successfully acquired a clone that lost the plasmid p652-2 and the *mcr-1.1*-harboring plasmid p652-4. The corresponding colistin resistance level of the plasmid-curing clone was reduced by more than 16 times. For many other resistance determinants carried on these four plasmids, plasmid acquisition or loss accompanying the change in resistance level in various derivate clones was mostly coincident with the predicted phenotype of antimicrobial agents mediated by the resistance determinants (**Figure 1**).

### Characterization of *mcr-1.1*- and *mcr-8.2*-harboring plasmids

**Figure 2:**
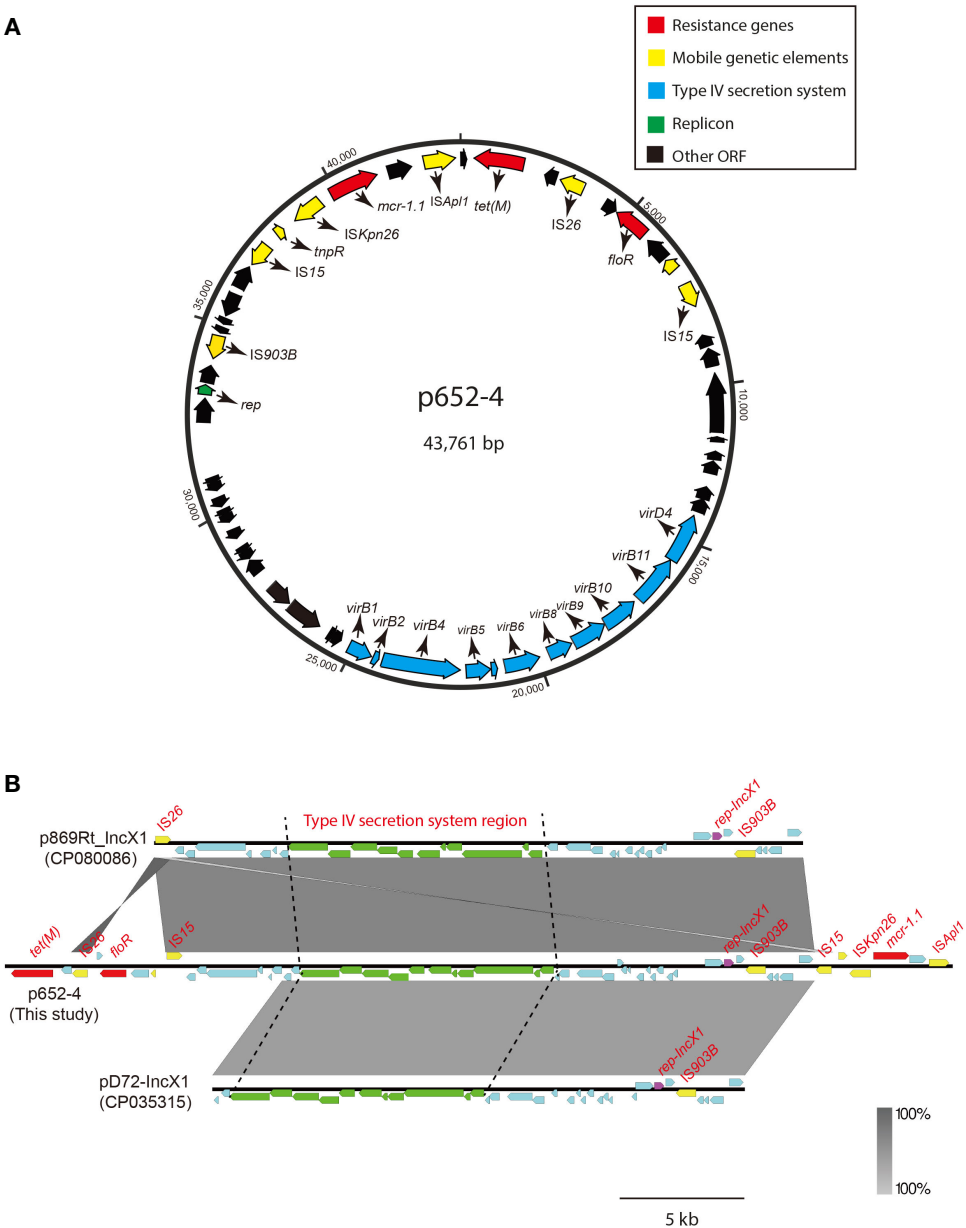
Circular map of plasmid p652-4 harboring *mcr-1.1* and comparison with other IncX1 plasmids. **(A)** Blue arrows indicate the replicon. Red arrows indicate antimicrobial resistance genes. Yellow arrows represent IS elements. The blue arrows indicate the *vir* operon that plays a role in the Type IV secretion system (T4SS). The black arrows indicate other open reading frames (ORFs). **(B)** Plasmid comparison with p869Rt_IncX1 (CP080086) and pD72-IncX1 (CP035315). Near 100% identical sequences are bridged by gray shading. Red filled boxes indicate resistance genes. Yellow filled boxes indicate insertion sequences (ISs). Purple filled boxes indicate replicons. Green filled boxes represent the T4SS region.

The *mcr-1.1* gene in KP15-652 is located on the approximately 44-kb plasmid named p652-4, with 44.57% GC content and belonging to IncX1. Structurally, the genetic environment of IS*Kpn26*-*mcr-1.1*-*pap2*-IS*Apl1* was observed (**Figure 2A**). In addition to *mcr-1.1*, the tigecycline efflux pump gene *tet*(M) was identified on p652-4, elevating the level of tigecycline resistance. Moreover, plasmid p652-4 was found to carry type IV secretion system-related genes due to a complete *vir* operon, which is identical to several known plasmids, such as p869Rt_IncX1 (CP080086) and pD72-IncX1 (CP035315) (**Figure 2B**). Hence, the sole plasmid p652-4 has the potential to be transferred *via* conjugation.

**Figure 3:**
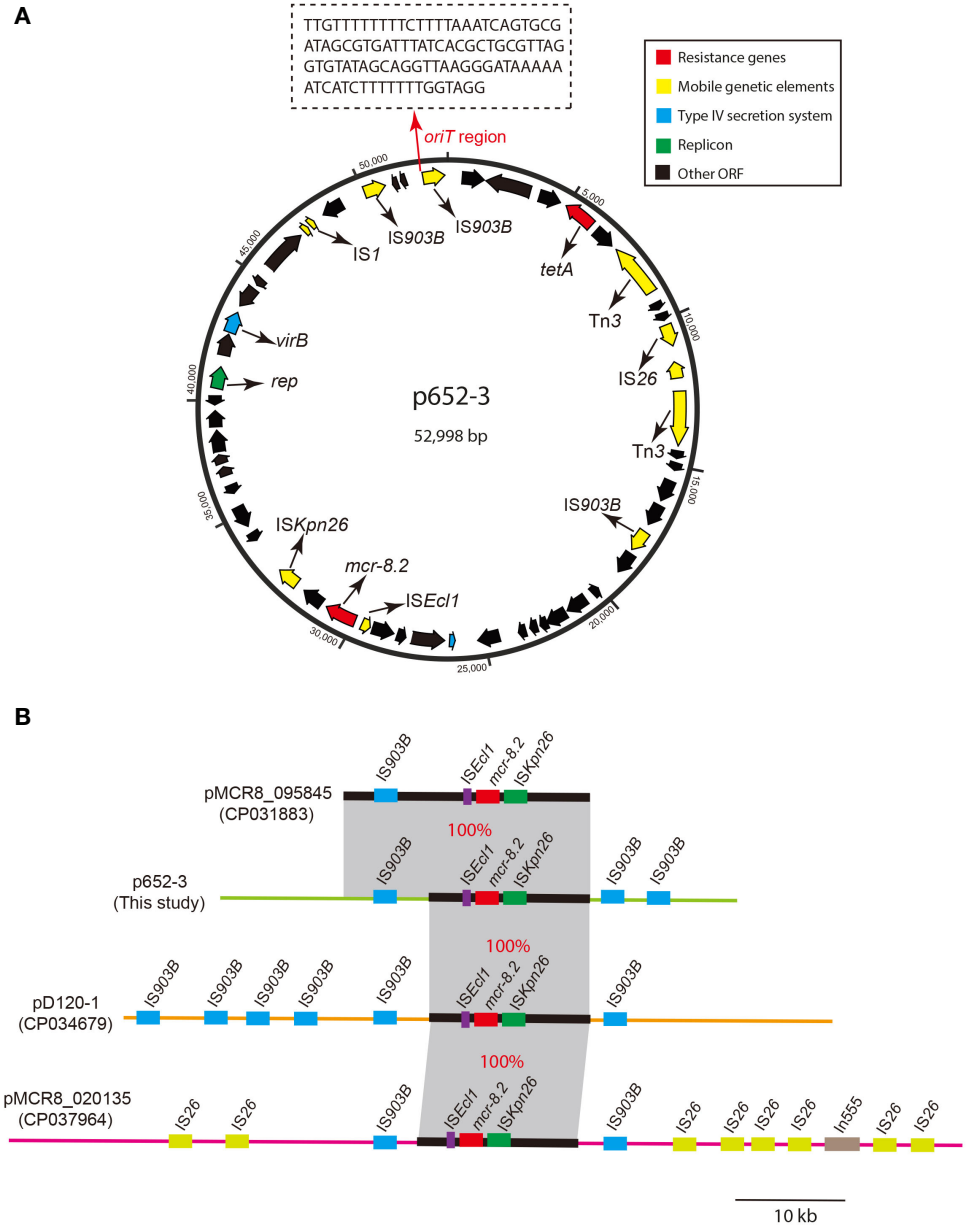
Circular map of plasmid p652-3 harboring *mcr-8.2* and plasmid comparison with pMCR8_095845, pD120-1 and pMCR8_020135. **(A)** Circular map of the p652-3 plasmid. Replicon (green), antimicrobial resistance genes (red), IS elements (yellow), T4SS (blue) and other ORFs (black) are shown. Another thin red arrow indicates the *oriT* region for the origin of transfer. **(B)** Linear structure of the p652-3 plasmid compared with pMCR8_095845 (CP031883), pD120-1 (CP034679) and pMCR8_020135 (CP037964). Near 100% identical sequences are bridged by gray shading. Red filled boxes indicate *mcr-8.2* genes. Blue, green, and purple filled boxes represent IS*903B*, IS*Kpn26* and IS*Ecl1*, respectively. Eight IS*26* copies are also shown in pMCR8_020135 in light green.

The *mcr-8.2* gene is located on the multireplicon plasmid p652-3 with IncR/IncN-typed replicons. The genetic structure of the *mcr-8.2* surrounding region of the p652-3 plasmid is IS*Ecl1*-*mcr-8.2*-*orf*-IS*Kpn26* (**Figure 3A**) and 100% identical to pMCR8_095845 (CP031883), pD120-1 (CP034679) and pMCR8_020135 (CP037964) (**Figure 3B**). Another tigecycline efflux pump gene, *tet*(A), was identified on the p652-3 plasmid, and the p652-3, pMCR8_095845, pD120-1 and pMCR8_020135 plasmids carry one or more IS*903B* genetic elements. No transferability system has been found for p652-3, with unsuccessful transfer of a single plasmid in the conjugation assay.

### Characterization of the *tmexCD1-toprJ1*-harboring plasmid

**Figure 4:**
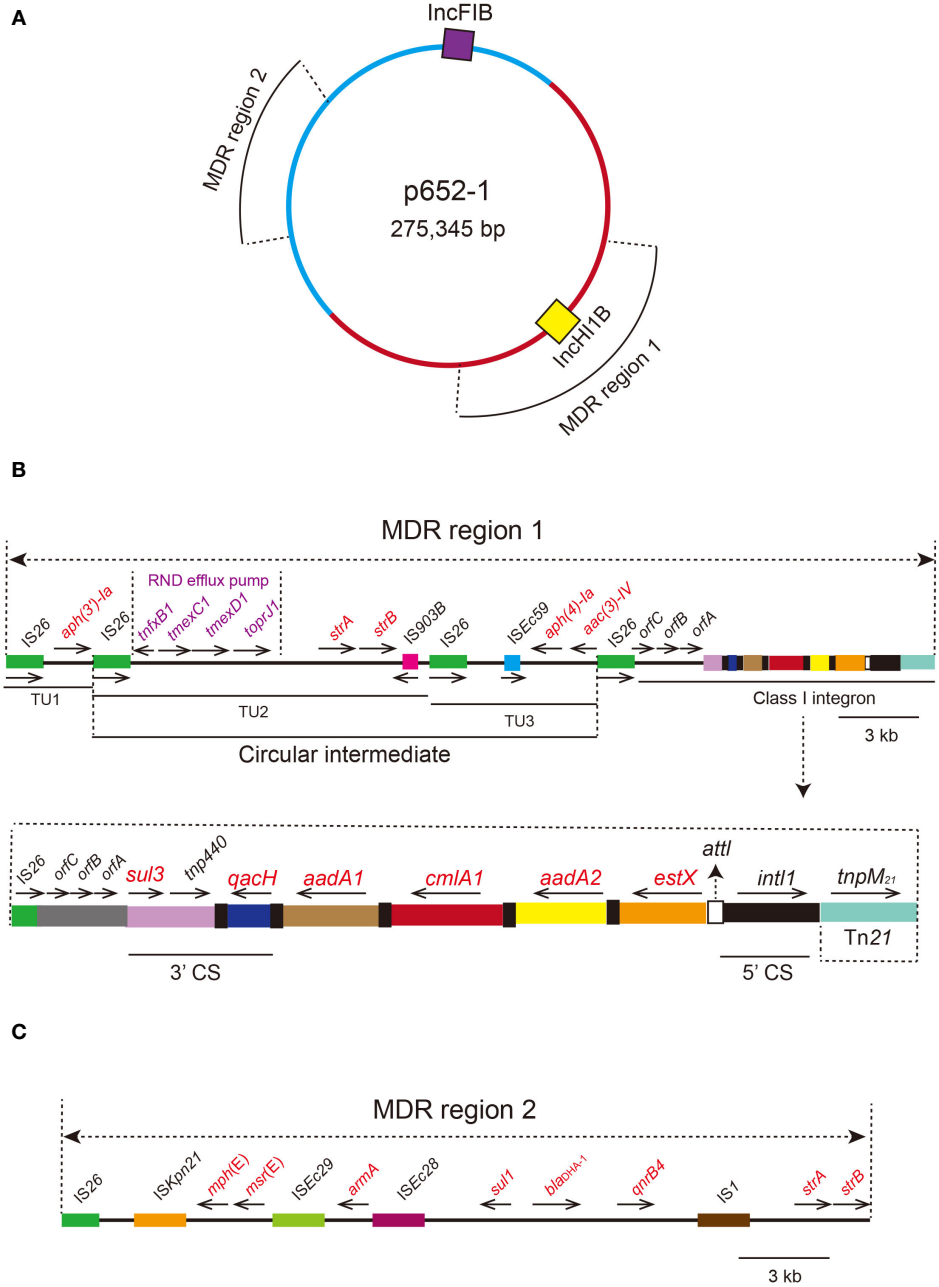
Circular proposed map of plasmid p652-1 harboring the *tmexCD1-toprJ1* gene cluster and the genetic structure of two multidrug resistance (MDR) regions. **(A)** Violet- and yellow-filled boxes indicate IncFIB and IncHI1B replicons, respectively. Two MDR regions are also shown in different positions. **(B)** The genetic structure of MDR region 1. IS*26* copies are shown as green filled boxes, with black arrows indicating the direction. A circular intermediate form of the *tmexCD1-toprJ1* gene cluster is shown. Resistance genes are labeled red in the circular intermediate form. The 5’ conserved segment (5’ CS) and 3’ CS of the class 1 integron are labeled. Various kinds of resistance genes in the integron are also shown as different colors and the names labeled above with the orientation indicated by thin black arrows. **(C)** The genetic structure of MDR region 2. IS elements are shown as colored boxes. Various kinds of resistance genes are labeled with red.

Plasmid p652-1 contains two multidrug resistance (MDR) regions, and the *tmexCD1-toprJ1* gene cluster is located in MDR region 1 (**Figure 4**). Many mobile genetic elements were also identified in MDR region 1, including four copies of IS*26*, a single copy of IS*903B*, *tnp440* and a partial Tn*21* (**Figure 4B**). A circular intermediate form of the *tmexCD1-toprJ1* gene cluster was observed, comprising two translocatable unit (TU) elements bracketed by two IS*26* elements. Importantly, a complex class 1 integron is located in the same region, consisting of a 5’ conserved segment (5’ CS) and 3’ CS (**Figure 4B**). Furthermore, various resistance genes, including *aadA2*, *cmlA1*, *aadA1*, *qacH* and *sul3*, were detected, and a segment of the transposase Tn*21* was identified downstream of *intl1*. MDR region 2 also harbors various genes conferring resistance to different kinds of antimicrobial agents, including *strA*, *strB*, *qnrB4*, *bla*_DHA-1_, *sul1*, *armA*, *msr*(E) and *mph*(E) (**Figure 4C**).

### IS*26*-mediated co-integration in plasmid p652-1

**Figure 5:**
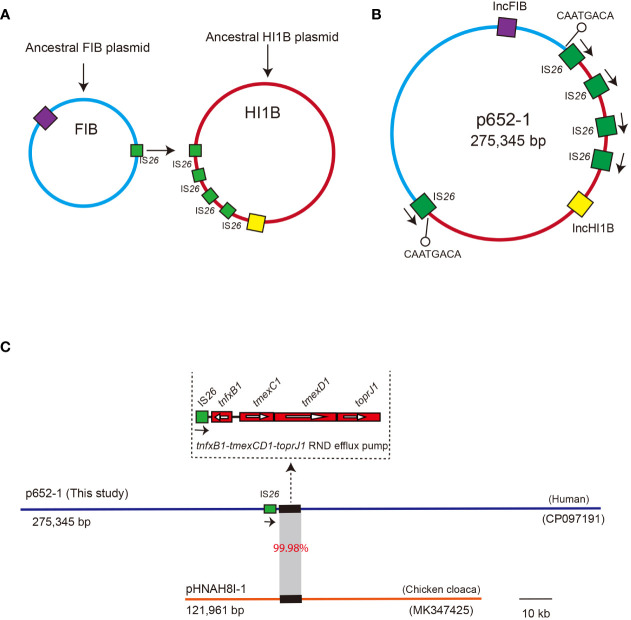
Proposed diagram for the formation of the co-integrate p652-1 plasmid. **(A)** The ancestral IncFIB plasmid and IncHI1B plasmid. Replicons are shown as purple and yellow filled boxes, respectively. IS*26* copies are shown as green filled boxes. **(B)** A co-integrate containing IncFIB-IncHI1B sequences *via* an IS*26*-mediated transposition event. Positions of the 8-bp target site duplication (TSD) adjacent to two IS*26* are indicated by a black line with a hollow circle. Directions of IS*26* are indicated *via* black arrows. **(C)** Plasmid comparison between p652-1 (this study, CP097191) and pHNAH8I-1 (MK347425). Plasmid backbones are shown as thick, blue and red lines, with one copy of IS*26* shown as green filled boxes with directions indicated by black arrows. Thick lines indicate the *tmexCD1*-*toprJ1* RND efflux pump-encoding gene cluster. Gray shading indicates the region with 99.98% identity.

The 275,345 bp plasmid p652-1 might be a co-integration plasmid derived from the progenitor IncHI1B-type plasmid and IncFIB-type plasmid. It is possible that the *tmexCD1-toprJ1* gene cluster is located on the IncHI1B-type plasmid, and the fusion process for its derivation may have involved IS*26*-mediated replicative transposition, whereby one single copy of IS*26* on the IncFIB-type plasmid targeted the other copy of IS*26* on the IncHI1B-type plasmid to form a fusion plasmid (**Figure 5A**). This event generated the 8-bp target site duplication (TSD) CAATGACA (**Figure 5B**). However, one IS*26* copy seemed to have undergone the inversion event according to the direction of IS*26* copies and the 8-bp TSD position.

We further conducted a comparison with the plasmid pHNAH81-1 (MK347425), a representative plasmid that harbors an RND efflux pump conferring resistance to tigecycline. The results showed only 6% coverage, with 99.92% identity between the p652-1 and pHNAH81-1 plasmids. Specifically, the *tmexCD1-toprJ1* gene cluster shows 99.98% identity and 100% coverage with that of the pHNAH81-1 plasmid. Moreover, one single copy of IS*26* was identified upstream of *tmexCD1-toprJ1* in p652-1. Nevertheless, no IS*26* genetic element was found on the pHNAH81-1 plasmid (**Figure 5C**).

## Discussion

Colistin and tigecycline are usually active *in vitro* and *in vivo* against MDR microorganisms, especially carbapenemase-producing *K. pneumoniae*, in critically ill patients (Karaiskos and Giamarellou, 2014). In general, the emergence of colistin- and tigecycline-resistant *K. pneumoniae* strains is alarming, with only few options for treatment. Here, we report a clinically isolated ST656 XDR-KP named KP15-652, which presented high-level colistin resistance mediated by multiple determinants, including *mcr-1.1*, *mcr-8.2* and *crrB* mutations (Liu et al., 2016; Jayol et al., 2017; Wang et al., 2018), combined with tigecycline resistance induced by a series of efflux pump gene, such as *tmexCD1-toprJ1*, *tet*(A) and *tet*(M) (Fiedler et al., 2016; Lv et al., 2020; Xu et al., 2021). These resistance determinants, combined with many other genes conferring resistance to multiple drug classes, are chromosome-located or plasmid-mediated. The majority of the acquired resistance genes are encoded by several distinct plasmids, some of which have been integrated from different Inc group ancestral plasmids.

The extensive use of colistin in veterinary medicine and humans has led to the emergence of colistin resistance (Mmatli et al., 2020). As well documented, the underlying mechanisms contributing to resistance are mainly mediated *via* lipid A modifications involving mutations of genes encoding the two-component systems on the chromosome and plasmids harboring the *mcr*-type phosphoethanolamine transferase enzyme (Quan et al., 2017; Zhang et al., 2018). In the current study, the colistin resistance shown by clinical isolate KP15-652 was attributed to several determinants located both on the chromosome and plasmids. Each of them contributed a considerable level of colistin resistance, which was confirmed by transformation/conjugation tests or plasmid curing assays. Plasmid-mediated *mcr-1.1* and *mcr-8.2* are located on two separate plasmids. Acquisition of the *mcr-1.1*-harboring plasmid might elevate the colistin resistance level by more than 64 times, and loss of it would reduce the MIC by more than 16 times. Similarly, the colistin MIC would increase by more than 64 times if a recipient isolate acquired the *mcr-8.2*-harboring plasmid. The plasmid curing assay confirmed the colistin resistance contribution of the chromosome-located *crrB* gene mutation because the cured isolate had an MIC that was 32 times higher than that of the transformant, which exhibited the same plasmid pattern but lacked the original *crrB* mutation. On the basis of the genetic context for *mcr-8.2*-carrying plasmids, we suggest that IS*903B* might play an important role in the mobility of the IS*Ecl1*-*mcr-8.2*-*orf*-IS*Kpn26* region because two copies of IS*903B* are also located on both sides of the *mcr-8.2*-flanking regions on pD120-1 and pMCR8_020135 (Zhao et al., 2022). Consequently, we inferred that it is possible that the high-level colistin resistance observed was caused by the accumulated effect of several factors on the chromosome and *mcr*-type plasmids together.

Tigecycline resistance of the clinical isolate KP15-652 was also owing to several factors, most of which are related to the efflux pump system on either the chromosome or plasmid. Either *tet*(A) or its mutated gene would typically be responsible for the increased tigecycline resistance that has been reported previously (Xu et al., 2021). Furthermore, *tet*(M) was able to elevate the tigecycline MIC to a moderate level, which was verified in either the transformant or conjugant under tigecycline selection. The efflux pump *tmexCD1-toprJ1*, which has only been well described in recent years, confers resistance in clinical isolates because acquisition of the *tmexCD1-toprJ1*-harboring plasmid led to elevated 256 times of tigecycline MICs compared to the recipient control.

Both plasmid fusion and mobile genetic elements (MGEs), including ISs, integrons and various transposons, play important roles in the resistance gene transfer (He et al., 2022). In these distinct MGEs, IS*26* produces a TU element which enables a transfer of a single IS*26* copy and adjacent DNA segment to a new position, contributing to DNA segment deletion or inversion through a replicative route (Harmer and Hall, 2015; Harmer and Hall, 2016). Based on sequence data, we found that tigecycline resistance may be caused by co-existence of *tet*(A), *tet*(M) and the *tmexCD1-toprJ1* gene cluster (Lv et al., 2020; Sun et al., 2020). Regarding the origin and transfer mechanisms of the *tmexCD1-toprJ1* gene cluster, we consider two possible reasons for acquiring the segment containing the *tmexCD1-toprJ1* gene cluster by horizontal gene transfer: transfer of the *tmexCD1-toprJ1* gene cluster *via* a circular intermediate composed of three copies of IS*26* and relevant DNA segments between IS*26* elements (Wan et al., 2021); and one copy of IS*26* inserted upstream of *tmexCD1-toprJ1* to form a TU, followed by the transfer event. The reason why we propose the second hypothesis is that there is 99.98% identity in the *tmexCD1-toprJ1* gene cluster between the p652-1 and pHNAH81-1 plasmids, and no IS*26* was detected upstream on the latter (Lv et al., 2020). Thus, we speculate that the IS*26* element located upstream of *tmexCD1-toprJ1* led to an insertion event.

In summary, this study describes a clinically isolated ST656 *K. pneumoniae* isolate that harbors high level colistin resistance determinants combined with several efflux pumps responsible for tigecycline resistance. Most of these extensively drug-resistant determinants are located on distinct plasmids, which can be transferred and influenced by their resistance phenotype. IS*26*-mediated plasmid fusion and MGE are of great importance in the transferability and evolution of resistance genes. Therefore, reasonable monitoring should be strengthened to prevent further spread of such colistin- and tigecycline-resistant *K. pneumoniae* in the healthcare setting.

## Data availability statement

The datasets presented in this study can be found in online repositories. The names of the repository/repositories and accession number(s) can be found below: https://www.ncbi.nlm.nih.gov/genbank/, CP097190-CP097197.

## Author contributions

YW and JZ contributed equally in this study. YJ and YY designed the study. YW and HL collected the isolates. JXZ, QW, PZ, JYZ, DZ, and XW analyzed and interpreted the data. YW and YJ wrote the manuscript. All authors contributed to the article and approved the submitted version.

## References

[B1] Beabout K. Hammerstrom T. G. Perez A. M. Magalhaes B. F. Prater A. G. Clements T. P.. (2015). The ribosomal S10 protein is a general target for decreased tigecycline susceptibility. Antimicrob. Agents Chemother. 59 (9), 5561–5566. doi: 10.1128/AAC.00547-15 26124155 PMC4538488

[B2] Carattoli A. Zankari E. Garcia-Fernandez A. Voldby Larsen M. Lund O. Villa L.. (2014). In silico detection and typing of plasmids using PlasmidFinder and plasmid multilocus sequence typing. Antimicrob. Agents Chemother. 58 (7), 3895–3903. doi: 10.1128/AAC.02412-14 24777092 PMC4068535

[B3] Dong N. Zeng Y. Wang Y. Liu C. Lu J. Cai C.. (2022). Distribution and spread of the mobilised RND efflux pump gene cluster tmexCD-toprJ in clinical gram-negative bacteria: a molecular epidemiological study. Lancet Microbe 3 (11), e846–e856. doi: 10.1016/S2666-5247(22)00221-X 36202114

[B4] El-Mansi M. Anderson K. J. Inche C. A. Knowles L. K. Platt D. J. (2000). Isolation and curing of the klebsiella pneumoniae large indigenous plasmid using sodium dodecyl sulphate. Res. Microbiol. 151 (3), 201–208. doi: 10.1016/s0923-2508(00)00140-6 10865947

[B5] El-Sayed Ahmed M. A. E. Zhong L. L. Shen C. Yang Y. Doi Y. Tian G. B. (2020). Colistin and its role in the era of antibiotic resistance: An extended review 2000-2019). Emerg. Microbes Infect. 9 (1), 868–885. doi: 10.1080/22221751.2020.1754133 32284036 PMC7241451

[B6] Fiedler S. Bender J. K. Klare I. Halbedel S. Grohmann E. Szewzyk U.. (2016). Tigecycline resistance in clinical isolates of enterococcus faecium is mediated by an upregulation of plasmid-encoded tetracycline determinants tet(L) and tet(M). J. Antimicrob. Chemother. 71 (4), 871–881. doi: 10.1093/jac/dkv420 26682961

[B7] Gao X. Wang C. Lv L. He X. Cai Z. He W.. (2022). Emergence of a novel plasmid-mediated tigecycline resistance gene cluster, tmexCD4-toprJ4, in klebsiella quasipneumoniae and enterobacter roggenkampii. Microbiol. Spectr. 10 (4), e0109422. doi: 10.1128/spectrum.01094-22 35862955 PMC9431256

[B8] Gorrie C. L. Mirceta M. Wick R. R. Judd L. M. Lam M. M. C. Gomi R.. (2022). Genomic dissection of klebsiella pneumoniae infections in hospital patients reveals insights into an opportunistic pathogen. Nat. Commun. 13 (1), 3017. doi: 10.1038/s41467-022-30717-6 35641522 PMC9156735

[B9] Harmer C. J. Hall R. M. (2015). IS26-mediated precise excision of the IS26-aphA1a translocatable unit. mBio 6 (6), e01866–e01815. doi: 10.1128/mBio.01866-15 26646012 PMC4676283

[B10] Harmer C. J. Hall R. M. (2016). IS26-mediated formation of transposons carrying antibiotic resistance genes. mSphere 1 (2), e00038-16. doi: 10.1128/mSphere.00038-16 PMC4894685 27303727

[B11] He J. Du X. Zeng X. Moran R. A. van Schaik W. Zou Q.. (2022). Phenotypic and genotypic characterization of a hypervirulent carbapenem-resistant klebsiella pneumoniae ST17-KL38 clinical isolate harboring the carbapenemase IMP-4. Microbiol. Spectr. 10 (2), e0213421. doi: 10.1128/spectrum.02134-21 35225687 PMC9045192

[B12] Jayol A. Nordmann P. Brink A. Villegas M. V. Dubois V. Poirel L. (2017). High-level resistance to colistin mediated by various mutations in the crrB gene among carbapenemase-producing klebsiella pneumoniae. Antimicrob. Agents Chemother. 61 (11). doi: 10.1128/AAC.01423-17 PMC5655078 28874377

[B13] Jin X. Chen Q. Shen F. Jiang Y. Wu X. Hua X.. (2021). Resistance evolution of hypervirulent carbapenem-resistant klebsiella pneumoniae ST11 during treatment with tigecycline and polymyxin. Emerg. Microbes Infect. 10 (1), 1129–1136. doi: 10.1080/22221751.2021.1937327 34074225 PMC8205050

[B14] Karaiskos I. Giamarellou H. (2014). Multidrug-resistant and extensively drug-resistant gram-negative pathogens: Current and emerging therapeutic approaches. Expert Opin. Pharmacother. 15 (10), 1351–1370. doi: 10.1517/14656566.2014.914172 24766095 PMC4819585

[B15] Linkevicius M. Sandegren L. Andersson D. I. (2016). Potential of tetracycline resistance proteins to evolve tigecycline resistance. Antimicrob. Agents Chemother. 60 (2), 789–796. doi: 10.1128/AAC.02465-15 26596936 PMC4750697

[B16] Liu Y. Lin Y. Wang Z. Hu N. Liu Q. Zhou W.. (2021). Molecular mechanisms of colistin resistance in klebsiella pneumoniae in a tertiary care teaching hospital. Front. Cell Infect. Microbiol. 11. doi: 10.3389/fcimb.2021.673503 PMC8576191 34765565

[B17] Liu Y. Y. Wang Y. Walsh T. R. Yi L. X. Zhang R. Spencer J.. (2016). Emergence of plasmid-mediated colistin resistance mechanism MCR-1 in animals and human beings in China: A microbiological and molecular biological study. Lancet Infect. Dis. 16 (2), 161–168. doi: 10.1016/S1473-3099(15)00424-7 26603172

[B18] Liu B. Zheng D. Zhou S. Chen L. Yang J. (2022). VFDB 2022: a general classification scheme for bacterial virulence factors. Nucleic Acids Res. 50 (D1), D912–D917. doi: 10.1093/nar/gkab1107 34850947 PMC8728188

[B19] Lv L. Wan M. Wang C. Gao X. Yang Q. Partridge S. R.. (2020). Emergence of a plasmid-encoded resistance-Nodulation-Division efflux pump conferring resistance to multiple drugs, including tigecycline, in klebsiella pneumoniae. mBio 11 (2), e02930-19. doi: 10.1128/mBio.02930-19 32127452 PMC7064769

[B20] Mmatli M. Mbelle N. M. Maningi N. E. Osei Sekyere J. (2020). Emerging transcriptional and genomic mechanisms mediating carbapenem and polymyxin resistance in enterobacteriaceae: a systematic review of current reports. mSystems 5 (6), e00783-20. doi: 10.1128/mSystems.00783-20 PMC7771540 33323413

[B21] Poirel L. Jayol A. Nordmann P. (2017). Polymyxins: Antibacterial activity, susceptibility testing, and resistance mechanisms encoded by plasmids or chromosomes. Clin. Microbiol. Rev. 30 (2), 557–596. doi: 10.1128/CMR.00064-16 28275006 PMC5355641

[B22] Quan J. Li X. Chen Y. Jiang Y. Zhou Z. Zhang H.. (2017). Prevalence of mcr-1 in escherichia coli and klebsiella pneumoniae recovered from bloodstream infections in China: a multicentre longitudinal study. Lancet Infect. Dis. 17 (4), 400–410. doi: 10.1016/S1473-3099(16)30528-X 28139430

[B23] Siguier P. Perochon J. Lestrade L. Mahillon J. Chandler M. (2006). ISfinder: the reference centre for bacterial insertion sequences. Nucleic Acids Res. 34 (Database issue), D32–D36. doi: 10.1093/nar/gkj014 16381877 PMC1347377

[B24] Sullivan M. J. Petty N. K. Beatson S. A. (2011). Easyfig: a genome comparison visualizer. Bioinformatics 27 (7), 1009–1010. doi: 10.1093/bioinformatics/btr039 21278367 PMC3065679

[B25] Sun J. Chen C. Cui C. Y. Zhang Y. Liu X. Cui Z. H.. (2019). Plasmid-encoded tet(X) genes that confer high-level tigecycline resistance in escherichia coli. Nat. Microbiol. 4 (9), 1457–1464. doi: 10.1038/s41564-019-0496-4 31235960 PMC6707864

[B26] Sun S. Gao H. Liu Y. Jin L. Wang R. Wang X.. (2020). Co-Existence of a novel plasmid-mediated efflux pump with colistin resistance gene mcr in one plasmid confers transferable multidrug resistance in klebsiella pneumoniae. Emerg. Microbes Infect. 9 (1), 1102–1113. doi: 10.1080/22221751.2020.1768805 32401163 PMC8284978

[B27] Tatusova T. DiCuccio M. Badretdin A. Chetvernin V. Nawrocki E. P. Zaslavsky L.. (2016). NCBI prokaryotic genome annotation pipeline. Nucleic Acids Res. 44 (14), 6614–6624. doi: 10.1093/nar/gkw569 27342282 PMC5001611

[B28] Wan M. Gao X. Lv L. Cai Z. Liu J. H. (2021). IS26 mediates the acquisition of tigecycline resistance gene cluster tmexCD1-toprJ1 by IncHI1B-FIB plasmids in klebsiella pneumoniae and klebsiella quasipneumoniae from food market sewage. Antimicrob. Agents Chemother. 65 (3), e02178-20. doi: 10.1128/AAC.02178-20 33361297 PMC8092543

[B29] Wang C. Z. Gao X. Yang Q. W. Lv L. C. Wan M. Yang J.. (2021a). A novel transferable resistance-Nodulation-Division pump gene cluster, tmexCD2-toprJ2, confers tigecycline resistance in raoultella ornithinolytica. Antimicrob. Agents Chemother. 65 (4), e02229-20. doi: 10.1128/AAC.02229-20 33495220 PMC8097428

[B30] Wang Z. Li H. Zhang J. Wang X. Zhang Y. Wang H. (2021c). Identification of a novel plasmid-mediated tigecycline resistance-related gene, tet(Y), in acinetobacter baumannii. J. Antimicrob. Chemother. 77 (1), 58–68. doi: 10.1093/jac/dkab375 34634801

[B31] Wang Q. Peng K. Liu Y. Xiao X. Wang Z. Li R. (2021b). Characterization of TMexCD3-TOprJ3, an RND-type efflux system conferring resistance to tigecycline in Proteus mirabilis, and its associated integrative conjugative element. Antimicrob. Agents Chemother. 65 (7), e0271220. doi: 10.1128/AAC.02712-20 33875423 PMC8218640

[B32] Wang X. Wang Y. Zhou Y. Li J. Yin W. Wang S.. (2018). Emergence of a novel mobile colistin resistance gene, mcr-8, in NDM-producing klebsiella pneumoniae. Emerg. Microbes Infect. 7 (1), 122. doi: 10.1038/s41426-018-0124-z 29970891 PMC6030107

[B33] Wareth G. Neubauer H. (2021). The animal-foods-environment interface of klebsiella pneumoniae in Germany: an observational study on pathogenicity, resistance development and the current situation. Vet. Res. 52 (1), 16. doi: 10.1186/s13567-020-00875-w 33557913 PMC7871605

[B34] Wick R. R. Judd L. M. Gorrie C. L. Holt K. E. (2017). Unicycler: Resolving bacterial genome assemblies from short and long sequencing reads. PLoS Comput. Biol. 13 (6), e1005595. doi: 10.1371/journal.pcbi.1005595 28594827 PMC5481147

[B35] Wyres K. L. Cahill S. M. Holt K. E. Hall R. M. Kenyon J. J. (2020a). Identification of acinetobacter baumannii loci for capsular polysaccharide (KL) and lipooligosaccharide outer core (OCL) synthesis in genome assemblies using curated reference databases compatible with kaptive. Microb. Genom 6 (3), e000339. doi: 10.1099/mgen.0.000339 32118530 PMC7200062

[B36] Wyres K. L. Lam M. M. C. Holt K. E. (2020b). Population genomics of klebsiella pneumoniae. Nat. Rev. Microbiol. 18 (6), 344–359. doi: 10.1038/s41579-019-0315-1 32055025

[B37] Xu J. Zhu Z. Chen Y. Wang W. He F. (2021). The plasmid-borne tet(A) gene is an important factor causing tigecycline resistance in ST11 carbapenem-resistant klebsiella pneumoniae under selective pressure. Front. Microbiol. 12. doi: 10.3389/fmicb.2021.644949 PMC7943888 33717043

[B38] Yang X. Dong N. Liu X. Yang C. Ye L. Chan E. W.. (2021). Co-Conjugation of virulence plasmid and KPC plasmid in a clinical klebsiella pneumoniae strain. Front. Microbiol. 12. doi: 10.3389/fmicb.2021.739461 PMC8606748 34819921

[B39] Zankari E. Hasman H. Cosentino S. Vestergaard M. Rasmussen S. Lund O.. (2012). Identification of acquired antimicrobial resistance genes. J. Antimicrob. Chemother. 67 (11), 2640–2644. doi: 10.1093/jac/dks261 22782487 PMC3468078

[B40] Zhang H. Zhao D. Shi Q. Quan J. Li X. Yu Y. (2018). Mcr-1 gene has no effect on colistin resistance when it coexists with inactivated mgrB gene in klebsiella pneumoniae. Microb. Drug Resist. 24 (8), 1117–1120. doi: 10.1089/mdr.2017.0291 29768099

[B41] Zhao J. Li Z. Zhang Y. Liu X. Lu B. Cao B. (2022). Convergence of MCR-8.2 and chromosome-mediated resistance to colistin and tigecycline in an NDM-5-Producing ST656 klebsiella pneumoniae isolate from a lung transplant patient in China. Front. Cell Infect. Microbiol. 12. doi: 10.3389/fcimb.2022.922031 PMC9310643 35899054

